# Myelodysplastic Syndromes Diagnosis: What Is the Role of Molecular Testing?

**DOI:** 10.1007/s11899-015-0270-5

**Published:** 2015-07-01

**Authors:** Rafael Bejar

**Affiliations:** Division of Hematology and Oncology, Moores Cancer Center, University of California, San Diego, 3855 Health Sciences Drive MC 0820, La Jolla, CA 92093-0820 USA

**Keywords:** Myelodysplastic syndrome, Cytopenic patients, Clonal cytopenias, Somatic mutations

## Abstract

Diagnosing a myelodysplastic syndrome (MDS) can be challenging. Somatic mutations are common in MDS and might have diagnostic utility in patients with idiopathic cytopenias of undetermined significance (ICUS). However, using mutations to diagnose MDS is complicated by several issues: (1) no gene is mutated in most cases, (2) no mutated gene is highly specific for MDS, (3) clonal hematopoiesis is common in older individuals without disease, and (4) we lack outcome data for ICUS patients with clonal cytopenias of undetermined significance (CCUS). Despite these caveats, genetic sequencing can inform the diagnosis of MDS. CCUS patients more closely resemble patients with MDS than age matched controls with somatic mutations. Genetic testing can identify alternative diagnoses in cytopenic patients and help risk stratify those with proven MDS. While we cannot include somatic mutations in the diagnostic definition of MDS now, testing to recognize CCUS will help characterize outcomes in these diagnostically challenging patients.

## Introduction

For several of reasons, myelodysplastic syndromes (MDS) are often challenging to diagnose. MDS are heterogeneous disorders that share key clinical features, including ineffective clonal hematopoiesis, morphologic dysplasia, peripheral blood cytopenias, and a variable risk of transformation to acute myeloid leukemia (AML) [1]. However, clinical presentations can vary dramatically from patient to patient. Establishing the diagnosis of MDS requires the quantification of morphologic features such as dysplasia and bone marrow blast proportion, both of which are subject to interobserver variability even among expert hematopathologists [2, 3]. Diagnostic features of MDS are also frequently encountered in related disorders ranging from aplastic anemia (AA) and myeloproliferative neoplasms (MPN) to AML with myelodysplasia. Making an accurate diagnosis has important clinical consequences [4–6]. A more objective mechanism for diagnosing MDS, particularly in confounding cases, would be of great clinical benefit [6–8].

Hematologists have long incorporated molecular genetic information into the diagnostic evaluation of patients with myeloid diseases. In chronic myelogenous leukemia (CML), measurement of the leukocyte alkaline phosphatase has been replaced by detection of the *BCR*-*ABL* fusion transcript—the defining criterion for this disorder [9, 10]. Several subtypes of AML are defined by the presence of specific chromosomal translocations regardless of bone marrow blast proportion [11]. And, in the10 years since the discovery of *JAK2* mutations in MPNs, these lesions can be diagnostic of polycythemia vera (PV), essential thrombocythemia (ET), or primary myelofibrosis (PMF) in the appropriate clinical contexts [12]. We have learned as much about the molecular genetic basis of MDS in the last decade [13•]. There are well over 40 somatically mutated genes seen recurrently in MDS, one or more of which can be identified in over 90 % of patients [14••, 15••, 16••]. We understand how mutations are associated with many disease features like ring sideroblasts, cytopenias, monocytosis, and chromosomal abnormalities. The independent prognostic value of many recurrently mutated genes has been validated in numerous studies, and mutations that act as biomarkers of response to specific therapies have been described [17, 18, 19•, 20•]. Somatic mutations are not only indicative of clonal hematopoiesis, a defining feature of MDS, they identify the molecular drivers responsible for its pathogenesis. These facts, and our experience with diagnostic molecular tests in related myeloid disorders, suggest that mutations could readily be incorporated into the diagnostic criteria for MDS [21, 22].

Unfortunately, diagnostic utility of somatic mutations in MDS is complicated by several issues. These include concerns about poor specificity, the range of genetic variability present in MDS, and a poor understanding about the implications of mutations in patients who do not meet current diagnostic criteria. This article will review current diagnostic methods for MDS and will explore the role of molecular genetic testing in this context, focusing on its challenges and how these may be overcome.

## Section I—Current Diagnostic Criteria for MDS

**Fig. 1:**
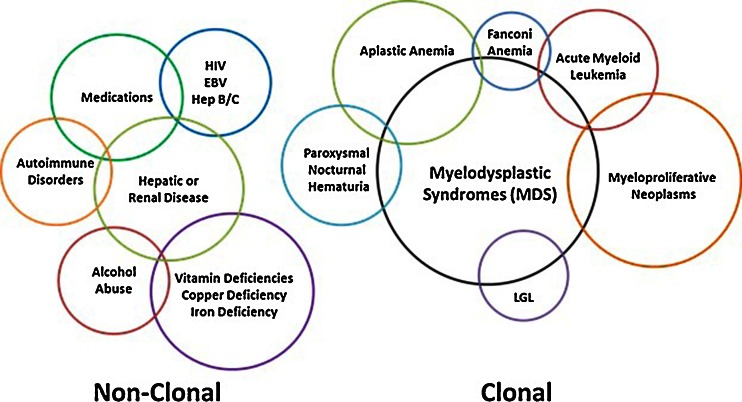
Diagnostic overlap between MDS and other clonal disorders and benign conditions that can mimic MDS. *HIV* human immunodeficiency virus, *EBV* Epstein-Barr virus, *Hep* hepatitis, *LGL* large granular lymphocyte leukemia

Making a diagnosis of MDS appears fairly straightforward. A patient must have at least one clinically meaningful cytopenia and a bone marrow examination with one or more “decisive” criteria for MDS (Table 1) [23]. However, meeting these criteria is not sufficient until competing explanations for these findings are excluded [11]. The list of benign conditions that can mimic MDS or confound its diagnosis is long (Fig. 1). Deficiencies of vitamin B_12_ or folate can cause cytopenias, megaloblastic changes, and macrocytosis. Iron deficiency can cause anemia and abnormal red cell morphology. Copper deficiency, often seen in patients with gastric bypass or chronic zinc ingestion, can result in anemia with ring sideroblasts, a defining feature of some MDS subtypes. Viral infections can cause bone marrow suppression with dysplastic features as can autoimmune conditions. These include Felty syndrome, idiopathic thrombocytopenia purpura, and systemic lupus erythematosus among others. Medications taken to treat autoimmune conditions, like methotrexate or azathioprine, can also cause cytopenias and dysplasia. Chronic alcohol abuse can cause morphologic dysplasia and cytopenias through a variety of mechanisms such as liver damage, splenomegaly, and direct bone marrow suppression. Then, there are several rare inherited conditions that can mimic MDS, such as congenital dyserythropoietic or sideroblastic anemias, which should be considered, particularly in cases with a family history of anemia. Finally, several clonal conditions that share clinical features with MDS, including AML, MPN, and AA, must also be excluded in order to make the diagnosis (Fig. 1).

**Table 1:** Diagnostic criteria for MDS and common findings

Peripheral blood findings	Bone marrow findings	Chromosomal abnormalities considered presumptive evidence of disease
One or more of the following:	And one or more of the following:	Translocations:
Hemoglobin <11 g/dL	≥10 % dysplasia in the granulocytic, erythroid, or megakaryocytic lineage	t(11;16)(q23;p13.3) t(2;11)(p21;q23)
Absolute neutrophil count <1500/μl (1.5 x 10^9^/L)	Myeloblasts comprise 5–19 % of total cellularity	inv(3)(q21q26.2) t(3;21)(q26.2;q22.1)
Platelet count <100,000/μl (100 x 10^9^/L)	Presence of an acquired chromosomal abnormality specific for MDS	t(1;3)(p36.3;q21.2) t(6;9)(p23;q34)
Commonly observed features:	Commonly observed features:	Abnormal copy number:
Neutrophil hypogranularity	Hypercellularity	−7 or del(7q)
Hypolobulated neutrophil nuclei (e.g., pseudo Pelger-Huët cells)	Nuclear-cytoplasmic asynchrony Karyorrhexis	−5 or del(5q) i(17q) or t(17p)
Monocytosis (in CMML)	Irregular nuclear contours	−13 or del(13q)
Immature leukocytes	Ring sideroblasts	del(12p) or t(12p)
Macrocytosis	Hypolobated megakaryocytes	del(9q)
Anisopoikilocytosis	Micromegakaryocytes	del(11q)
Hypochromic erythrocytes	Abnormal leukocyte granulation	idic(X)(q13)
Large or hypogranular platelets	Abnormal localization of immature precursors	Complex karyotype (3 or more abnormalities)
	Ectopic antigen expression by flow cytometry	
	Mild to moderate reticulin fibrosis

The WHO guidelines recognize the challenge of diagnosing patients with unexplained cytopenias, no increased blast proportion, and a normal karyotype [11]. They recommend that before making the diagnosis of MDS, patients with unilineage dysplasia as their sole diagnostic finding demonstrate 6 months of persistent cytopenia. Recovery of counts in that time would suggest a cause other than MDS. The presence of an acquired chromosomal abnormality can be helpful as it indicates clonal hematopoiesis. This does not exclude benign causes of cytopenias but might make it more likely that MDS or another clonal disorder is present. The WHO guidelines include a list of chromosomal abnormalities that can serve as presumptive evidence of the diagnosis, even in the absence of dysplasia or increased blast proportion (Table 1). These include several of the most frequent cytogenetic abnormalities seen in MDS such as del(5q), del(7q), loss of chromosome 7, or a complex karyotype (defined as the presence of three or more concurrent abnormalities). It is important to note that several recurrent chromosomal abnormalities are not included in this list. Lesions such as del(20q) and trisomy 8 are found in MDS but are present in many other myeloid disorders and hence lack the necessary specificity required to serve as diagnostic evidence of MDS. Loss of chromosome Y is also not considered a diagnostic clonal marker because it can frequently be seen in older men in the absence of hematologic disease. In practice, less than 50 % of patients with MDS will have an abnormal karyotype, and for lower risk patients with no increasing in blasts, this fraction is closer to 25 % [24, 25]. Cytopenic patients who do not reach the arbitrary cutoffs of 10 % dysplasia any lineage or 5 % bone marrow blast proportion will rarely carry a disease-defining chromosomal abnormality. In contrast, somatic mutations indicative of clonal hematopoiesis can be found in over 80 % of MDS patients with lower risk disease [26]. This begs the question of whether certain somatic mutations can substitute for chromosomal abnormalities as presumptive evidence of MDS in the absence of disease-defining morphologic criteria.

For reasons that are addressed below, the short answer to this question at the moment is no. Somatic mutations should not replace our current diagnostic standards for MDS as they have for several myeloproliferative disorders.

## Section II—Challenges to the Diagnostic Utility of Somatic Mutation in MDS

To understand why somatic mutations are not reliably diagnostic of MDS, it will be helpful to review the features of good diagnostic biomarkers. The first is frequency. A mutation present in the majority of cases of given disease is extremely useful for its diagnosis. Even if the mutation occurs in other disorders, the clinical context can help exclude these alternative diagnoses as unlikely. If a diagnosis is suspected on clinical grounds but its highly frequent, associated mutation is absent, the diagnosis must be considered much less likely or ruled out altogether. This describes *JAK2* mutations in the diagnosis of PV. Even though *JAK2* mutations can be found in a variety of myeloid disorders, including MDS, a mutation should be present in a patient clinically suspected of having PV. If no *JAK2* mutation can be identified after appropriate testing, an alternative diagnosis such as secondary or congenital polycythemia must be given greater weight as the diagnosis of PV is essentially excluded.

The second feature that can support the utility of a diagnostic biomarker is specificity. A mutation uniquely associated with a disease can be considered presumptive evidence of its diagnosis. This is true even if the majority of the patients with the disorder lack this particular mutation. Consider *MPL* mutations which are present in 5 % of ET. While these mutations are not present in the majority of ET patients, they are extremely rare in other conditions. Clinical context can exclude confounding conditions such as MF and refractory anemia with ring sideroblasts (RARS) and thrombocytosis (RARS-T). Therefore, a typical *MPL* mutation in a patient with isolated thrombocytosis strongly supports the diagnosis of ET. Several other MPN-associated gene mutations, such as those in *CALR* for PMF and ET, *KIT* in mastocytosis, and the *FIPL1*-*PDGFRA* rearrangement in the hypereosinophilic syndrome, can also be considered specific enough for diagnostic use in the appropriate clinical contexts. It is also helpful if the molecular lesion in question is the founding abnormality that gives rise to the disease. This implies a degree of specificity but also ensures that the lesion will be present in the majority of clonal cells making easier to detect. The *BCR*-*ABL* fusion gene in CML is the classic example of causative, disease-defining mutation that meets both the frequency and the specificity criteria described above, making it an excellent diagnostic marker.

The examples above are MPNs where acquired mutations are routinely incorporated into diagnostic criteria [11]. There are several reasons why this is not yet the case for MDS. To begin with, the clinical context for MDS is much more challenging to interpret than it is for MPNs. There are a limited number of molecular mechanisms that cause myeloproliferation and alternative diagnoses for patients with elevated cell counts are few and relatively rare. In contrast, cytopenias and myelodysplasia, the defining features of MDS, can be caused by a broad array of molecular abnormalities and many potential alternative causes of these findings are frequent. There is no single gene that is mutated in the majority of cases of MDS. Mutations of *TET2* and *SF3B1* are each present in only a quarter to a third of patients [15••, 16••, 26]. A handful of other genes (*ASXL1*, *SRSF2*, *DNMT3A*, and *RUNX1*) are mutated in 10–20 %, but the majority of recurrently mutated genes identified in MDS are present in fewer than 5 % of cases. Therefore, no mutated gene meets the frequency criteria that would allow a diagnosis of MDS to be excluded if a particular mutation was not identified.

Mutations could still be diagnostically useful if they were highly specific for MDS. Unfortunately, this is not the case. Like the del(20q) and trisomy 8 chromosomal abnormalities, many genes mutated in MDS are observed in other myeloid disorders where they have very different clinical implications. For example, *ASXL1* mutations are more common in chronic myelomonocytic leukemia (CMML) than in MDS but can also occur in MPN, AA, and AML [27–29]. Mutations of *TET2* and *DNMT3A* are found in these myeloid disorders but can occur in lymphoid neoplasms as well [30]. Mutations of *SF3B1* are the most specific as they are strongly associated with the presence of ring sideroblasts, a readily identifiable morphologic feature used to make the diagnosis of MDS [31•]. However, *SF3B1* mutations can also occur in MPN/MDS overlap syndromes like CMML and RARS-T and are frequent in CLL [32, 33].

Finally, there is a practical consideration that distinguishes the mutations that support the diagnosis of MPN and AML from the mutations most often seen in MDS. Many AML- or MPN-related mutations are highly recurrent activating lesions such as *JAK2* V617F, the *MPL* W515L/K mutations, internal tandem duplications of *FLT3*, or the frameshifts in the terminal exons of *CALR* and *NPM1*. Most of the somatic mutations in MDS are not so stereotypic. Genes can often be mutated anywhere along their length and can harbor missense mutations that can be difficult to distinguish from benign germline polymorphisms or incidental passenger mutations unrelated to the disease. This makes the interpretation of genetic tests more difficult for many MDS-related genes given our current state of knowledge.

Were these the only issues constraining the use of mutations as diagnostic biomarkers for MDS, certain mutations in specific genes might still have obvious diagnostic utility in the appropriate clinical context. For example, if a patient with ICUS (clinically significant cytopenias, morphologic criteria for MDS not met, and no alternative diagnosis evident) were found to have a typical mutation in *DNMT3A*, the presence of clonal hematopoiesis would be established potentially making a diagnosis of MDS more likely than a non-clonal cause of their cytopenias. However, recent findings dictate caution in making such an interpretation.

It is known that clonal hematopoiesis can occur in the absence of a clinically evident hematologic disorder. Studies of skewed X chromosome inactivation demonstrate that some women have clonal hematopoiesis and that this becomes increasingly likely with age [34, 35]. More recent work has confirmed that this is not a rare phenomenon [36]. In two studies, subjects participating in genome-wide association studies (GWAS) had their blood genotyped for single nucleotide polymorphisms (SNPs) [37, 38]. This technique found that 2–3 % of older participants carried chromosomal abnormalities likely to be acquired and therefore indicative of clonal hematopoiesis. Two subsequent GWAS studies and a study of patients with solid tumors examined the exomes of blood samples from a total of over 30,000 individuals [39••, 40, 41••]. Likely somatic mutations in several MDS-associated genes were found with a frequency that increased markedly with age. Approximately 10 % of persons aged 70–80 carried one or more of these mutations indicative of clonal hematopoiesis. The three most commonly mutated genes across these studies were *DNMT3A* (accounting for more than half of all cases), *TET2*, and *ASXL1* which were closely followed by mutations in *JAK2*, *TP53*, *SF3B1*, *SRSF2*, and *CBL*. Only one frequently mutated gene, *PPM1D*, was not known to be recurrently mutated in MDS [41••]. The median age of MDS patients is in the 70–75 years range. The incidence of MDS in this age group is several orders of magnitude lower that the 10 % rate of clonal hematopoiesis detected in this population. More importantly, most of the individuals with clonal hematopoiesis had no known hematologic abnormality and there was no association between the presence of somatic mutations in these GWAS studies and clinically significant cytopenias.

The presence of a somatic mutation in these studies was not clinically benign, however. Individuals with evidence of clonal hematopoiesis had a 10–15-fold increased risk of developing a hematologic malignancy, although not necessarily MDS [39••, 41••]. This risk increased to 50-fold if the somatic mutation was present in 20 % or more nucleated blood cells (corresponding to a variant allele fraction of ≥0.10). Yet, the absolute increase in risk remained very small, and the vast majority of patients with clonal hematopoiesis never went on to develop a hematologic disorder. For this reason, incidental findings of somatic mutations have been referred to as *clonal hematopoiesis of indeterminate potential*, abbreviated as CHIP [42••]. The high frequency of CHIP in the elderly population most likely to have MDS places strong constraints on the diagnostic value of somatic mutations. The presence of a *DNMT3A*, *ASXL1*, or *TET2* mutation typical of MDS does not clarify the diagnosis in an elderly cytopenic patient. Benign causes of cytopenias and alternative clonal disorders would still have to be excluded just as they would if these mutations were not present.

## Section III—Utility of DNA Sequencing at the Time of Diagnosis

Given the many caveats discussed above, it may appear that molecular genetic tests have no role in the diagnostic setting for MDS. However, there are several scenarios in which somatic mutations can clarify the diagnosis and, in the future, might inform a molecular definition of disease. CHIP was described in various populations that were not suspected of having a hematologic disorder. In practice, these are not the people subjected to molecular genetic testing. Instead, it is the cytopenic patients for which MDS is in the differential diagnosis that are more likely to undergo sequencing of their blood or bone marrow. Finding a somatic mutation typical for MDS in this clinical context may have different implications. Currently, patients with unexplained cytopenias that do not meet the diagnostic criteria for MDS are labeled as having ICUS [7, 43•, 44]. The eventual outcome for patients with ICUS is not well understood nor have clear risk features for progressive disease been established. Small studies have suggested that clonality may be one adverse risk factor [45]. Recent studies have demonstrated that a subset of ICUS patients carry somatic mutations in MDS-related genes indicative of clonal hematopoiesis [46, 47]. This may not seem surprising given the high rate of CHIP in the age range typical for MDS. However, ICUS patients with clonal hematopoiesis, henceforth referred to as having *clonal cytopenias of undetermined significance* (*CCUS*), have other features that distinguish them from people with non-cytopenic CHIP [42••].

Two studies presented at the American Society of Hematology Annual Meeting in 2014 examined diagnostic material from cytopenic patients suspected of having MDS. One was a prospective examination of 146 patients [46]. Based on their bone marrow findings, 21 patients were diagnosed with MDS, 22 as ICUS with some dysplastic features not meeting criteria for MDS, and 103 as having no morphologic features of MDS at all. A panel of 21 frequently mutated MDS genes was examined by next-generation sequencing in each patient. In patients with a clear diagnosis of MDS, 76 % had at least one typical somatic mutation with more than a third having three or more such mutations. The mutation rate was 55 % in the ICUS group with some evidence of MDS and 22 % in cytopenic patients with no evidence of MDS at all. The fraction of CCUS patients across the two ICUS arms was 28 %, which is much higher than the 10–15 % of CHIP expected in an aged matched “normal” population. Strikingly, the number of CCUS patient was nearly 1.5-fold greater than the number of patients that met the diagnostic criteria for MDS, suggesting that the incidence of CCUS may be large and comparable to that of MDS under its current diagnostic definition.

The second study examined 250 sequential cases of ICUS collected over 6 months at a commercial laboratory and compared them to 90 cases of lower risk MDS diagnosed in the same time frame [47]. The results were similar to the prior study in that 33 % of ICUS patients carried at least one somatic mutation. In both studies, the genes mutated in ICUS and MDS were similar with the exception of *SF3B1* which was rarely mutated in CCUS and common in MDS, particularly lower risk MDS. Interestingly, the median variant allele frequencies for MDS and CCUS were similar at about 30 %. More than 80 % of mutations were present at an allele frequency of at least 10 %, the median described for CHIP in population studies. The likelihood of having clonal hematopoiesis in a cytopenic patient was greater than the background rate of CHIP, and mutations were more abundant on average suggesting a continuum between CHIP, CCUS, and MDS. Unfortunately, we still lack data on outcomes in patients with CCUS and how clinical and molecular features could help us further refine disease risk in this group.

In absence of validated outcomes data, a Bayesian approach could help support the diagnosis of clinically meaningful cytopenias even if they are not labeled as MDS. For example, persons with CHIP were mostly over age 50 and typically had one MDS-related mutation at a low (<10 %) variant allele frequency. If a 45 year-old patient with ICUS were found to carry two or more mutated genes with a variant allele frequency of 30–40 %, it would seem much less likely that this person had CHIP and coincidentally, an overlooked benign cause for their cytopenias. Similarly, if a 73 year-old cytopenic patient has no somatic mutations detected in a large panel of genes, alternatives to MDS should be strongly considered.

We may learn that certain mutations have different degrees of diagnostic utility. For example, *DNMT3A*, *TET2*, and *ASXL1* are so frequent in CHIP and so prevalent in disorders other than MDS that an isolated mutation in one of these genes likely cannot be considered diagnostically helpful (Table 2). Some of the less frequently mutated CHIP genes, like *U2AF1*, *RUNX1*, and *TP53*, may retain some specificity for MDS in the appropriate clinical context.

**Table 2:** Frequency of recurrent MDS-associated mutations

	Gene	Typical mutations	Frequency in MDS (%)	Frequency in other hematologic malignancies	Frequency in CHIP (%)	Notes
Splicing factors	*SF3B1*	Heterozygous missense mutations at specific hotspots	18–35	Rare except for RARS-T (85 %) but also found in CLL (15 %)	4	Strongly associated with ring sideroblasts. Considered prognostically favorable in MDS.
	*SRSF2*	Heterozygous missense mutations at specific hotspots	10–15	More common in CMML (40 %)	2	Considered prognostically adverse in MDS.
	*U2AF1*	Heterozygous missense mutations at specific hotspots	8–12	Rare in AML (3 %)	<1	Considered prognostically adverse in MDS.
	*ZRSR2*	Premature stop codons or frameshifts	5–10	Very rare in AML (<1 %)	<1	On X chromosome—most often in found in men.
Epigenetic regulators	*TET2*	Premature stop codons, frameshifts, or missense in C terminal catalytic domains	20–25	Common in AML, but also found in MPN and lymphoid malignancies	10	Often bialellic or at site of aUPD.
	*DNMT3A*	Premature stop codons, frameshifts, or missense at codon R882	12–18	More common in AML (25 %), but also found in MPN and lymphoid malignancies	60	Maybe prognostically adverse in MDS.
	*IDH1* / *IDH2*	Heterozygous missense mutations at specific hotspots	2–5	More common in AML (20 %)	<1	Maybe prognostically adverse in MDS.
	*ASXL1*	Premature stop codons or frameshifts	15–25	More common in CMML (40 %) and also present in MPN and AML	10	Typically monoallelic. Considered prognostically adverse in MDS.
	*EZH2*	Premature stop codons, frameshifts, or missense in C terminal catalytic domain	5–10	More common in CMML (12 %)	<1	Often bialellic or at site of aUPD. Considered prognostically adverse in MDS.
Transcription factors	*RUNX1*	Premature stop codons, frameshifts, or missense in RUNX DNA-binding domain	10–15	Common in AML but also found in advanced MPN	NR	Maybe germline in rare cases. Considered prognostically adverse in MDS.
	*GATA2*	Missense mutations in GATA zinc finger domains	<1	Very rare	NR	Often germline if present.
	*ETV6*	Premature stop codons or frameshifts	<5	Similar in AML	<1	Maybe germline in rare cases. Considered prognostically adverse in MDS.
	*TP53*	Premature stop codons, frameshifts, or missense other that P47S and P72R	8–12	Similar in AML, but also found in MPN and lymphoid malignancies	3	Maybe germline in rare cases. Considered prognostically very adverse in MDS.
Cohesins	*STAG2*	Premature stop codons or frameshifts	2–5	Rare in AML (4 %)	<1	
	*RAD21*	Premature stop codons or frameshifts	<2	Rare in AML (3 %)	<1	
	*SMC3*	Premature stop codons or frameshifts	<2	Rare in AML (4 %)	<1	
Growth factor signaling	*NRAS*/*KRAS*	Heterozygous missense mutations at specific hotspots	5–10	More common in CMML (15 %) and also present in AML (10 %)	1	Considered prognostically adverse in MDS. Often subclonal.
	*JAK2*	V617F missense mutation	2–5	Very common in MPN: PV (90 %), ET (40 %), and PMF (60 %). Rare in AML	6	Can be bialellic due to aUPD.
	*CBL*	Missense mutations in RING domain	<5	More common in CMML (20 %)	1–2	Can be bialellic due to aUPD.
	*CALR*	Frameshifts in terminal codon	<2	More common in MPN: PMF (30 %) and ET (30 %)	NR	
Others	*GNAS*/*GNB1*	Heterozygous missense mutations at specific hotspots	<2	Very rare	4	
	*NPM1*	Frameshifts in terminal codon	1–2	More frequent in AML (30 %)	NR	
	*SETBP1*	Heterozygous missense mutations at specific hotspots	<5	Much more frequent in CMML (30 %)	NR	

Several genes are mutated much more frequently in disorders that can mimic MDS. For example, a *BRAF* mutation in a cytopenic patient without marked dysplasia should prompt careful examination for features of hairy cell leukemia, a rare cause of cytopenias that nearly always carries an activating *BRAF* mutation [48]. Similarly, recurrent mutations of *STAT3* and *STAT5B* have been associated with clonal large granular lymphocyte leukemia, a disorder of T or NK cells that can present like MDS, but is treated very differently [49, 50]. Just as t(8;21), inv(16), t(16;16), and t(15;17) define specific subtypes of AML regardless of bone marrow blast proportion, there may be mutated genes that are de facto evidence of leukemia. These might include small *FLT3*-ITD mutant subclones or more abundant mutations of *IDH1*, *IDH2*, or *NPM1*, all of which are very rare in both CHIP and MDS compared with AML [51, 52].

Rare cytopenic patients may have germline mutations in genes associated with an increased risk of developing MDS or AML. The genes involved can include *RUNX1*, *CEBPA*, *GATA2*, *ETV6*, *DDX41*, *TERT*, *DKC1*, and *TP53*, among others [53–56]. Patients may have a suggestive family history, earlier age at diagnosis, and clinical manifestations specific to the congenital mutation involved. However, patients can present later in life with bone marrow failure as their only prominent clinical finding, and germline mutations can occur de novo in individuals with no relevant family history [57, 58]. Often, patients may have a prodromal stage of abnormal blood counts without clear evidence of MDS or AML. This is common in patients with germline *RUNX1*, *ETV6*, or *GATA2* mutations, for example [54, 55]. Genetic testing at diagnosis may identify these individuals earlier in the course of disease and help screen siblings if related donor allogeneic stem cell transplant is being considered.

Finally, DNA sequencing at the time of diagnosis may identify somatic mutations that help classify patients with traditionally defined MDS. Currently, only patients with isolated del(5q) are classified based on a genetic abnormality. These patients share a clinical phenotype including a more favorable prognosis and a striking response to treatment with lenalidomide [59]. However, there are likely similar phenotypic subtypes of MDS that might be defined by somatic mutations. The most evident are those patients with *SF3B1* mutations who commonly have ring sideroblasts [31•, 60, 61]. In the current schema, patients with >15 % ring sideroblasts, <5 % myeloblasts, and isolated erythroid dysplasia are assigned the RARS subtype. This threshold for ring sideroblasts is arbitrarily defined and has no biologic significance. It may be better to define a subtype based on *SF3B1* mutation status independent of ring sideroblast percentage [62]. Several studies suggest that this would create a more uniform subset of patients [63, 64•]. On the other end of the risk spectrum, patients with *TP53* mutations may comprise another MDS subtype. Patients with *TP53* mutations have fewer mutations in other genes and often carry multiple chromosomal lesions that can include del(5q), monosomy 7, or an abnormal chromosome 17 [14••, 19•, 20•, 65, 66]. Clinically, these patients are more likely to have thrombocytopenia, excess bone marrow blasts, and shorter overall survival. Mutations in other genes, or recurrent combinations of genes, may define additional clinically significant MDS subtypes in the future [15••, 16••].

## Conclusion

Current evidence does not support the use of somatic mutations as presumptive evidence of MDS absent traditional diagnostic criteria [42••]. However, genetic testing at the time of initial evaluation can aid in establishing a diagnosis and can provide additional clinically relevant information. In cases that meet morphologic criteria for MDS, typical somatic mutations strongly support the diagnosis. Several of these lesions have demonstrated prognostic significance that can refine risk stratification and impact treatment decisions. As with the del(5q) chromosomal abnormality, some gene mutations may be strongly associated with clinical features and thus be used to help classify MDS subtypes in patients that meet the classical diagnostic criteria.

In cytopenic cases that are diagnostically unclear, somatic mutations must be interpreted more cautiously [8]. Genetic mutations recurrently identified in MDS patients lack the frequency and specificity required to serve as presumptive evidence of the disease, particularly due to high rate of CHIP defined by the same abnormalities in patients without a hematologic disorder. Even in this setting, somatic mutations can be useful. Finding more than one typical mutation at higher allele frequencies may raise the likelihood that the mutations and cytopenias are linked, particularly in younger patients where the rate of incidental CHIP is low. Other mutations, if present, may raise the likelihood of diagnoses other than MDS or identify patients with a congenital mutation predisposing to MDS/AML. And, the absence of mutations in a sufficiently broad gene panel may identify patients with lower risk of progression or in which an eventual diagnosis of MDS is less likely.

The key to these approaches will be to start with a careful appreciation of the clinical context. Low frequency somatic mutations in patients with mild cytopenias and only marginal suspicion for MDS should be given little significance. In fact, these patients should likely not be tested for mutations at all. Patients with more profound cytopenias in which alternative diagnoses have been carefully explored may be more likely to harbor clinical relevant mutations. These CCUS patients may merit closer observation, or if severely affected, might be treated similarly to patients with lower risk, transfusion dependent MDS. As it stands today, there is insufficient evidence regarding the long-term outcomes of patients with CCUS to merit redefining the diagnostic criteria for MDS. With this in mind, an added benefit of sequencing cytopenic patients at the time of diagnosis is that it will allow us to recognize and study CCUS patients longitudinally to determine the impact of mutations on their eventual outcome.
